# Effect of *Baliospermum montanum* Root Extract on Phagocytosis by Human Neutrophils

**DOI:** 10.4103/0250-474X.51966

**Published:** 2009

**Authors:** Kalpana S. Patil, S. S. Jalalpure, R. R. Wadekar

**Affiliations:** Department of Pharmacognosy, K.L.E.S.'s College of Pharmacy, Belgaum-590 010, India

**Keywords:** Immunostimulant, *Baliospermum montanum*, nitroblue tetrazolium test, phagocytosis

## Abstract

Aqueous extract of roots of *Baliospermum montanum* was evaluated on preliminary basis for immunomodulatory activity by studying neutrophil phagocytic function. The different concentration of (25, 50, 100 μg/ml) of aqueous extract of roots of *Baliospermum montanum* was subjected to study its effect on different *in vitro* methods of phagocytosis such as neutrophil locomotion, chemotaxis, immunostimulant activity of phagocytosis of killed *Candida albicans* and qualitative nitroblue tetrazolium test by using human neutrophils. This preliminary study revealed that *Baliospermum montanum* extract has stimulated chemotactic, phagocytic and intracellular killing potency of human neutrophils at the different concentration. From the results obtained it can be observed that the aqueous extract of *Baliospermum montanum* stimulate cell-mediated immune system by increasing neutrophil function.

The immune system is known to be involved in the etiology as well as pathophysiologic mechanism of many diseases. Immunology is thus probably one of the most rapidly developing areas of biomedical research and has great promises with regard to prevention and treatment of wide range of disorders, inflammatory diseases of skin, gut, respiratory tract, joints and central organs. In addition infectious diseases are now primarily considered immunological disorders while neoplastic diseases and organ transplantation and several autoimmune diseases may involve in an immunosuppressive state[1].

The function and efficacy of the immune system may be influenced by many exogenous factors like food and pharmaceuticals, physical and psychological stress and hormones resulting in either immunostimulation or immunosuppression. The healthy state is believed to be based on a sophisticated fine-tuning of immunoregulatory mechanisms[2].

Suppressive and cytotoxic activity affecting the function of immune system has been reported in many of the synthetic and natural therapeutic agents. Among the synthetic substances, azathioprin and cyclophasphmide were alkylating agent resulting in the cross linking of DNA and causes inhibition of DNA synthesis. The major drawbacks of these drugs are myelosuppressive, which is undesirable. Immunomodulator of herbal origin appearing to be a better alternative to overcome the above problem[3].

*Baliospermum montanum* Muell Arg of family Euphorbiaceae is a stout under shrub with herbaceous branches from the roots[4]. It is found in tropical and subtropical Himalaya from Kashmir eastwards to Arunachal Pradesh. It is reported to contain axillarenic acid, baliospermin and montanin, which possess wide range of activities such as anthelmentic, diuretic, purgative, bronchitis[5]. The survey of literature reveals that the whole plant and roots of *Baliospermum montanum* are found to be used in traditional system of medicine as a tonic[4]. However, immunomodulatory activity of *Baliospermum montanum* has not been reported scientifically investigated.

Thus in the present study, an attempt has been made to evaluate immunomodulatory potency of aqueous extract of roots of *B. montanum* using different *in vitro* methods for locomotion, phagocytic and intracellular killing potency of neutrophil which are subsequent events involved in the process of phagocytosis by neutrophils.

The roots of *B. montanum* were collected from the local areas of Belgaum and authentified at the Botanical Survey of India, Koregaon, Pune. A voucher specimen (BSI/WC/Tech 272) of the plant material is kept at the Pharmacognosy museum of KLES's college of Pharmacy, Belgaum. The freshly collected roots of the plant were shade dried at room temperature and powered until able to pass through sieve number 40.

The powdered root was macerated with chloroform water IP 1996 for 6 days. The dark brown filtrate obtained was concentrated by heating on a water bath which was then lyophilized and stored at 4° until further use. The crude aqueous extract was subjected to phytochemical investigation.

Sample of 5% for *in vitro* study was prepared by dissolving 2.5 g of crude extract in 25 ml PBS (phosphate buffer solution pH 7.2) to obtain a solution of 100 μg/ml. From this stock solution, different working dilutions were prepared to get a concentration range of (25, 50, 100 μg/ml) neutrophils of the blood withdrawn from normal human volunteers were used to study the activity. PBS (pH 7.2) used as a vehicle. Neutrophil cell suspension was prepared in phosphate buffer solution with about 10^6^ cells/ml. The lower compartment of chemotactic chamber (5 ml beaker) was filled with appropriate chemotactic reagents preadjusted to pH of 7.2. e.g. chamber 1-PBS solution (control), chamber 2-casein 1 mg/ml (standard) and chambers 3, 4, 5 with different concentration (25, 50, 100 μg/ml) of test sample. The upper compartment (1 ml syringe) was filled with neutrophil cell suspension and wet filter (millipore) of 3 mm pore size was fixed at the bottom of the upper compartment. The upper compartment placed over the lower compartment incubate at 37° for 180 min.

The upper compartment was removed and inverted to empty the fluid. The lower surface of the filter was fixed with 70% ethanol for 2 min and then stained with heamatoxylin dye (Hi-media) for 5 min. The fixed filters were observed under microscope using 100 X lens and the number of neutrophils cells reached to the lower surface of the filter was counted.

*In vivo* immunomodulatry studies by slide method[6] the *Candida albicans* culture was incubated in Sabouraud broth overnight and then centrifuged to form a cell button at the bottom and supernatant was discarded. The cell button was washed with sterile Hanks Balanced Salt solution (HBSS) and centrifuged again. This was done 3-4 times. The final cell button was mixed with a mixture of sterile HBSS (Hi-media) and human serum in proportion of 4:1.The cell suspension of concentration 1×10^8^ was used for the experiment.

Human blood (0.2 ml) was obtained by finger prick method on a sterile glass slide and incubated at 37° for 25 min to allow clotting. The blood clot was removed very gently and slide was drained slowly with sterile normal saline, taking care not to wash the adhered neutrophil (invisible). The slide consisting of polymorphonuclear neutrophils (PMNS) was flooded with predetermine concentration of test sample and incubated at 37° for 15 min. The PMNS were covered with *Candida albicans* slide and incubated at 37° for 1 h. The slide was drained, fixed with methanol and stained with Giemsa stain (Hi-media). Positive control was tested by preparing the slide in a same way with pooled normal human serum.

The mean number of *Candida* cells phagocytosed by PMNS on the slide was determined microscopically for 100 granulocytes using morphological criteria. This number was taken as phagocytic index (PI) and was compared with basal PI of control. This procedure was repeated for different concentration (25, 50, 100 μg/ml) of test sample. Immunostimulation in % was calculated by using the equation, stimulation (%) = (PI(test)-PI (control)/PI (control) ×100.

A suspension of leucocytes (5×10^6^/ml) was prepared in 0.5 ml of PBS solution in 5 test tubes 0.1 ml of PBS solution (control) and 0.1 ml of endotoxin activated plasma (standard) is added to the 1^st^ and 2^nd^ tube, respectively and to the other 3 tubes of test sample 0.1 ml of different concentration (25, 50, 100 μg/ml) of test sample. 0.2 ml of freshly made 0.15% NBT solution was added to each tube and incubated at 37° for 20 min, centrifuged at 400 g for 3-4 min to discard the supernatant. The cells were resuspended in a small volume of PBS solution.

A thin film was made with the drop on a slide, dried and fixed by heating, counter stained by dilute carbol-fuschin for 15 sec. The slide was washed under tap water, dried and observed under 100 X oil emulsion objective. Two hundred neutrophils were counted for the % of NBT positive cells containing blue granules/lumps.

The values are expressed in mean±SEM. The results were analyzed by one way analysis of variance (ANOVA) followed by Dunnet's ‘t’ test to determine the statistical significance[7]. The level of significance was set at p<0.001.

The preliminary phytochemical investigation reveals presence of tannins, saponins, flavonoid and glycosides. The aqueous extract of roots of *B. montanum* has caused a significant increase (p<0.001) in movement of number of neutrophils from the upper compartment to lower surface of filter in a dose dependent manner (Table 1), stimulation of phagocytosis of *Candida albicans* by neutrophils (Table 2) and also increase in percentage of NBT positive cells containing the reduced NBT dye (Table 3), when compared with control samples containing PBS solution.

**TABLE 1:** EFFECT OF AQUEOUS EXTRACT OF ROOTS OF *BALIOSPERMUM MONTANUM* ON NEUTROPHIL LOCOMOTION AND CHEMOTAXIS

Groups	Concentration μg/ml	Mean number of neutrophil/field
Control (PBS)	-	5.60±0.71
Standard (Casein)	01	70.29±1.25*
*B. montanum* extract	25	46.25±1.30*
*B. montanum* extract	50	49.55±1.40*
*B. montanum* extract	100	52.21±1.49*

Each value is represented as mean±SEM of n=3;

* p<0.001 compared to control group.

**TABLE 2:** EFFECT OF AQUEOUS EXTRACT OF ROOTS OF *BALIOSPERMUM MONTANUM* ON NEUTROPHIL PHAGOCYTOSIS

Groups	Concentration μg/ml	Mean number of neutrophil/field
Control (Pooled Plasma Serum)	-	4.85±0.86
*B. montanum* extract	25	27.34±1.08*
*B. montanum* extract	50	29.12±1.22*
*B. montanum* extract	100	32.65±1.26*

Each value is represented as mean±SEM of n=3;

* p<0.001 compared to control group.

**TABLE 3:** EFFECT OF AQUEOUS EXTRACT OF ROOTS OF *BALIOSPERMUM MONTANUM* ON QUANTITATIVE NBT TEST

Groups	Concentration μg/ml	% NBT Positive Cells
Control (PBS)	-	21.30±1.05
Endotoxin-activated plasma	-	75.05±0.94
*B. montanum* extract	25	61.46±0.84*
*B. montanum* extract	50	65.36±1.17*
*B. montanum* extract	100	83.16±1.08*

Each value is represented as mean±SEM of n=3;

* p<0.001 compared to control group.

In neutrophil locomotion and chemotaxis test and qualitative NBT test, the results obtained with *B. montanum* were comparable with that of standard casein. Immunomodulatory agents of plant and animal origin increase the immune responsiveness of the body against pathogens by activating the non-specific immune system. However; there is a need to subject such medicinal plants to systematic studies to substantiate the therapeutic claims made with regard to their clinical utility[8,9].

Recently there is an enthusiasm towards exploration of novel group of compounds from natural sources that modulate the immune response of living systems and influence the disease process[10,11]. In the present study, aqueous extract of roots of *B. montanum* significantly (p<0.001) increased the phagocytic function of human neutrophils when compared to control indicating, the possible immunostimulating effect. The *B. montanum* extract has significantly increased the neutrophil chemotactic movement indicated by the increase in number of cells reaching the microorganism after slide method which provides a rapid and simple means of assessing the overall phagocytic process by the neutrophils.

The aqueous extract of roots of *B. montanum* has significantly increased in ingestion of *Candida albicans* by neutrophils. The aqueous extract of roots of *Baliospermum montanum* has significantly increased the intercellular reduction of NBT dye to formazen (deep blue compound) by the neutrophils, confirming the intracellular killing property of phagocytosing neutrophils.

From the results obtained, it can be concluded that the aqueous extract of roots of *Baliospermum montanum* has exhibited significant effect on phagocytosis by human neutrophils and chemotactic locomotion of neutrophils. Thus, the plant can be further explored for its phytochemical profile to identify the active constituents responsible for the above mentioned activities.
